# Accentuating the Interrelation between Consumer Intention and Healthy Packaged Food Selection during COVID-19: A Case Study of Pakistan

**DOI:** 10.3390/ijerph18062846

**Published:** 2021-03-11

**Authors:** Muhammad Zeeshan Zafar, Adnan Maqbool, Lucian-Ionel Cioca, Syed Ghulam Meran Shah, Shahjahan Masud

**Affiliations:** 1 Faculty of Management Sciences, University of Central Punjab, Lahore 54100, Pakistan; 2 Department of Management Sciences, Khwaja Fareed University of Engineering and Information Technology, Rahim YarKhan 64200, Pakistan; adnanpak@yahoo.com; 3 Department of Industrial Engineering and Management, Faculty of Engineering, Lucian Blaga University of Sibiu, Bd. Victoriei No. 10, 550024 Sibiu, Romania; 4 Academy of Romanian Scientists, 3 Ilfov Street, Sector 5, 010071 Bucharest, Romania; 5 School of Business Administration, South Western University of Finance and Economics, Chengdu 611130, China; ghulam_meeran2001@yahoo.co.in (S.G.M.S.); shahjahan.masud@hotmail.com (S.M.)

**Keywords:** COVID-19, attitude, subjective norms, self-efficacy, intention

## Abstract

This study contemplates the factors that influence consumer intention, before and during the eruption of COVID-19, for the selection of healthy packaged food in Pakistan. The extant studies have identified two distinct attitudes of consumers about food label information: one is its usefulness and the second elucidates the avoidance. Hence forth, the current study contributes to the extant literature while signifying both reasons which motivate consumers to read food labels and reasons which discourage consumers from consult food labels at the point of purchase. Moreover, the impact of subjective norms and self-efficacy for healthy packaged food intentions has also been examined for both before the emergence of COVID-19 and during the spread of COVID-19. The underpinning of the proposed model has been justified by the behavioral reasoning theory. The cross-sectional data of 14,455 students has was collected from 10 universities through Microsoft Teams and Zoom. AMOS 21 was employed for the final analysis. The results indicate that before COVID-19 the subjective norms and self-efficacy were not the stimulating factors for the selection of healthy packaged food. On the contrary, during the COVID-19 outbreak, the subjective norms and self-efficacy divulged a significant effect. Moreover, the reasons to consult food labels are positively significant whereas the reasons to avoid food labels have negatively affected the consumer, both before COVID-19 and during COVID-19 outbreak, while endorsing healthy packaged food. Conclusively, COVID-19 has been proved to be a deterrent for unhealthy packaged food lovers while being a blessing for healthy packaged food.

## 1. Introduction

The COVID-19 pandemic is substantially affecting lifestyles, national healthcare systems and global economies [1]. Social isolation is doubtlessly an unpleasant experience that may negatively influence mental health [2]. Explicitly, it is evident that diet has a profound effect on people’s immune systems and disease susceptibility [3]. Therefore, the key to maintain an effective immune system is to avoid deficiencies of the nutrients that play an essential role in immune cell triggering, interaction, differentiation, or functional expression [4]. 

Since to date there is no vaccine or evidence-based treatment for curing COVID-19 [5], the optimization of nutrient intake through well-balanced meals and the use of good hygiene practices in food selection, preparation, and conservation is probably the most encouraging approach for managing the continuous risk of viral infection [6]. To this end, the dissemination of healthy eating guidelines from healthcare professionals and the general public is a crucial strategy. Despite intense efforts by international nutrition organizations and other health-related societies to providing guidelines and advice to help control the COVID-19 pandemic, the literature is still scarce [7,8]. Meanwhile, the general public has been bombarded with a vast array of nutritional information from governmental authorities, the dietary supplement industry, nutrition enthusiasts and healthcare professionals emphasizing how to prevent COVID-19 [9]. Interestingly, a worldwide trend has observed showing that individuals are interested in stocking up on processed foods during quarantine [10]. 

Remarkably, during quarantine, a change in behavior while selecting food has been observed. Moreover, consumers prefer to select healthy packaged food; specifically, they select red meat, biscuits, and spreadable creams [11]. It is difficult to understand which factor stimulates consumers to select healthy packaged food. Additionally, numerous factors motivate the consumers to select healthy packaged food including food labels information [12], self-interest and the opinions of peers or family members [13]. The aforementioned studies have reported inconsistent results regarding the impact of various factors for the selection of healthy packaged food. Some of the studies have identified that food label formats convince consumers to select healthy packaged food at the point of purchase [14]. On the contrary, other studies have revealed the insignificant effect of food label information for healthy and nutritious packaged food selection [15]. Moreover, some literature has revealed the fact that an individual’s self-ability is vigorously interlinked with the selection of healthy packaged food at the time of purchase [16]. However, some results of prior studies have witnessed the insignificant effect of self-efficacy for the selection of healthy packaged food [17]. Henceforth, there is a dire need to examine the specific factors which cause this changed behavior [18]. 

Further, it is worthwhile to investigate the factors that influence and motivate consumers regarding healthy packaged food choices [19]. Accordingly, the prior studies have indicated that the display of nutritional information with various icons like a heart, leaf, or any other visual cues, also does not ensure consumers’ healthy packaged food selection [20]. Therefore, it is necessary to test hypotheses to examine the reasons that justify consumer’ intentions regarding the purchase of healthy packaged food items. The concept of reasons is itself dichotomous; it includes reasons for and reasons against [21], which have been examined among different research settings [22,23]. In the current study, the researchers have simultaneously investigated the impact of reasons which motivate and demotivate individuals’ consumption intentions regarding healthy packaged foods during COVID-19. The concept is underpinned with Behavioral Reasoning Theory (BRT) [24], which provides a linkage between beliefs, global motives (attitudes, subjective norms, perceived behavioral control), intentions, and heterogeneous behaviors. 

## 2. Literature

The extant literature has identified that front of pack labelling (FoPL), not only assists individuals with making informed decisions [25,26] but also encourages companies to improve their products concerning nutritional compositions. Although prior studies have reported that various forms of FoPL such as the green keyhole, traffic light symbols, and nutri-scores are getting popularity around the globe [27], nevertheless, some of the studies have reported the discrepancies among consumers’ favorable and unfavorable opinions towards the usefulness of FoPL at point of purchase for the selection of healthy packaged food products [28]. There is a need to contemplate these discrepancies for the generalized format of FoPL for informed decisions for healthy packaged food items [29]. Consumers’ abilities to employ detailed FoPL information does not ensure healthy food selection [30].

Many theorists have endeavored to comprehend the basic determinants of intention and behavior [31]. Fortunately, the behavioral intention model plays a decisive role for the insight into behavioral and intention determinants [32]. Moreover, behavioral intention theorists rely on the theory of reasoned action and the theory of planned behavior for demonstrating its fundamental determinants [33]. These two models enunciate that attitude, subjective norms and perceived behavioral control/self-efficacy predict individual intentions and behavior. Moreover, the behavioral intention model hypothesizes that the concepts of belief predict attitude, subjective norms and perceived behavioral control [34]. Additionally, the concepts of beliefs are the context of specific reasons which motivate and demotivate the individual towards any object [35]. The extant literature has referred to the novel consumer behavior model, behavioral reasoning theory, to examine the antecedents which can encourage and discourage consumer behavior and intentions towards any object [36]. Therefore, BRT describes reasons associated with the linkage between beliefs, reasons, attitude, subjective norms, self-efficacy/perceived behavioral control, intention and behavior. According to BRT, reasons assist the individual in justifying the actions which protect and promote the individual’s self-worth [37]. Henceforth, the authors of the current study have underpinned their model on BRT to examine consumer intentions towards healthy packaged food selection.

The prior studies have identified multiple reasons which motivate consumers at point of purchase to consult packaged food information for the selection of healthy packaged food [38]. Besides, some of the studies have highlighted the reasons which demotivate consumer from consulting food label information at the point of sale [39] while identifying the motivational and demotivational reasons to consult food label information at point of purchase based on the efficacy and inefficacy of food label information [40]. In this regard, the current study identifies some reasons which encourage consumer attitudes to read food label information for health and some reasons which discourage consumer attitudes to read food labels for healthy packaged food selection. Moreover, the current study also examines the dichotomous impact of reasons like the reasons which encourage consumer attitude and reasons which discourage consumer intention to select healthy packaged food.

Several researchers have adopted subjective norms and self-efficacy for behavioral intention prediction [41]. The scholars have conducted a meta-analysis of 161 studies and found that 39% of the variances in intention are accounted for by attitude, subjective norms and self-efficacy [42]. Along with that, it has also been observed that 2% out of 39% belong to self-efficacy. This is small but significant. Prior studies have also deployed attitude, subjective norm and self-efficacy for the investigation of breakfast intention among adolescents [43]. Significantly, the results have exposed the fact that 53.1% of the variance in intention has been accounted for by attitude and self-efficacy, whereas subjective norms remain insignificant. The dimensions of the behavioral intention model illuminate several health-related issues like intake of fat reduction [44] and eating behavior with respect to health [45]. Some authors have addressed the healthy eating intentions of individuals with subjective norms [46]. The results signify that 43% of the variance in healthy eating behavior has been accounted for by attitude, subjective norm and self-efficacy. Furthermore, scholars [47] have also investigated the effect of the behavioral intention model on the physical activity and healthy eating habits of individuals. Conclusively, the current study has examined the impact of subjective norms and self-efficacy on the selection of healthy packaged food.

During the spread of COVID-19, medical specialists and practitioners have recommended healthy food for individuals to improve their immune system. It was the most suitable time to investigate an individual’s opinions about their selection of packaged food. In this regard, the current study has employed front of pack label, subjective norm and self-efficacy to contemplate the specific factors which are catalytic for the selection of healthy packaged food before COVID-19 and during the eruption of COVID-19.

## 3. Methods

### 3.1. Questionnaires

Cross-sectional data collected with adapted questionnaire. Seven items were used for subjective norm and nine items were used for self-efficacy [48]. The authors of the study used [49] method to develop the questionnaire to measure the reasons which make up consumer attitudes to select healthy packages and the reasons which create hindrances for selecting healthy packaged food. Nine reasons were identified from the past studies to make up the attitudes for food labels and nine reasons were identified from the past studies to make up the attitudes against food labels. The intention to select healthy packaged food [50] was measured with seven items. All the questions were on five-point Likert scale. The measurements are available in detail in Appendix A.

### 3.2. Sample Size and Questionnaires Distribution

The researchers chose university students for the sample due to their consciousness of health and healthy food choices. University students tend not only to select healthy food items for themselves but also recommend such items to others; they are considered opinion leaders for the rest of society. The aforementioned studies involved university students to investigate their healthy eating behavior and intention [51]. There are 123 private and public sector universities in Pakistan, and the questionnaire was emailed to all department deans of all private and public sector universities for formal permission. Permission was granted by only 10 universities out of 123.The majority of universities did not grant permission due to their online classes. They mentioned that it is very difficult to manage any kind of informal activity during online sessions. Owing to the lockdown all students were engaged in taking classes on Microsoft Teams and Zoom. Therefore, the student affairs department uploaded the questionnaire on their portal. To achieve the objective of the current study, all students were instructed to record their opinions about their behavior before COVID-19 in the first study, and in the second questionnaire, although it contained the same questions, their answers were to be about their behavior during COVID-19.

### 3.3. Measurement Methods

Structural equation models (SEMs) were used for data analysis. The causal relations between the latent exogenous and latent endogenous variables were measured with a standard coefficient and significance value; for this purpose, AMOS 21 (IBM and SPSS Inc., Chicago, IL, USA) was used to run the measurement and path model. The adequate fit was observed in the present study by comparing it with standard fit indices [52]. An SEM was used to examine the factors which determine individuals’ intentions to consume packaged food items.

## 4. Theoretical Framework

**Figure 1:**
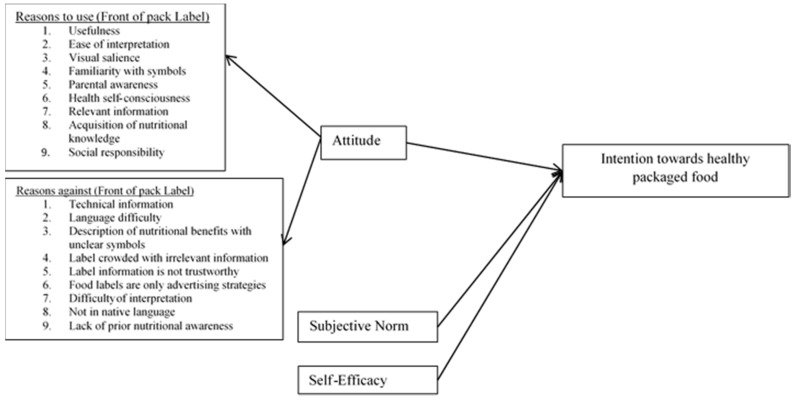
Healthy Packaged food intention in COVID-19.

Figure 1 represents the graphic relationship among all variables in the current study. The model was supported by BRT [53]. The inconsistent results of past studies regarding the attitude to consult food label information at point of purchase encouraged the authors of the current study to examine the reasons which influence consumer attitude and reasons which create barriers for consumers to consult label information at the point of purchase for healthy packaged food. Therefore, the authors of current study identified nine reasons which can motivate consumers to consult food label information for healthy packaged food and nine reasons which discourage consumers from consulting food labels for healthy packaged food selection. Moreover, consumer decisions towards the selection of any object are influenced by influential members of society like parents, friends, peers and other opinion leaders [54]. According to Ajzen these opinion leaders are called the “subjective norm” [55]. Therefore, the author of the study employed subjective norms to examine their role in the selection of healthy packaged food. Although there are multiple external factors which affect consumer decisions and intention, nevertheless, consumer self-decisions significant. Therefore, the current study involved the self-efficacy factor to examine its impact on consumer healthy packaged food selection intention.

### Hypothesis

**Hypothesis** **1** **(H1).** *Reasons that influence attitude to consult food label positively and significantly affected consumer intention to select healthy packaged food before and during COVID-19.*

**Hypothesis** **2** **(H2).** *Reasons that influence attitude to avoid food labels negatively and significantly affected consumer intention to select healthy packaged food before and during COVID-19.*

**Hypothesis** **3** **(H3).** *Subjective norms significantly affected the intention to select healthy packaged foods before and during COVID-19.*

**Hypothesis** **4** **(H4).** *Self-efficacy significantly affected the intention to select healthy packaged foods before and during COVID-19.*

## 5. Analysis

### 5.1. Description of Sample

The total received questionnaires were 18,355 from 10 different universities. During data entry 3788 questionnaires were discarded—usable questionnaires were 14,567. Of these 14,567 respondents, 6556 were females and 8012 were males. The imbalance in gender representation was notable, so an independent sample *t*-test was conducted. It was assumed that there was no difference between the genders in the selection of healthy packaged food items. The results revealed that the designed model was effective for both males and females. According to Hofstede, Pakistan is a collectivist society, so the respondents were asked to indicate their education, age, and parents’ incomes in a demographics section. The average age of the female respondents was 24, while the average age of the males was 25.

### 5.2. Analysis

Ninety-one questionnaires were missing less than 10% of data, so the imputation method was employed to replace the missing data. The suggested test for outlier detection is the Mahala Nobis distance [56], which identified 112 questionnaires as outliers. The researchers deleted them from the final data set. Ultimately, 14,455 questionnaires were included in the final analysis. Convergent and discriminate validity tests were conducted to check the content and constructs’ validity.

### 5.3. Convergent Validity

For the assessment of the convergent validity, standardized factor loading was linked with latent constructs. The convergent validity was measured with composite reliability [57]; the recommended cut-off value for composite reliability was pegged at 0.60, although some researchers have advocated that a cut-off value of 0.70 provides better reliability [28]. To ensure convergent validity, two other criteria were assessed; the value of the construct reliability, which should not be less than 0.70, and the variance extraction, which should be greater than 0.50 [58]. The results indicated that all constructs of the measurement model adequately demonstrated reliability and convergent validity.

### 5.4. Discriminate Validity

Discriminate validity was examined using average variance extraction (AVE). By rule of thumb, the value of the square of the correlation of two measured constructs should be less than the AVE [59]. Similarly, if the square root of the AVE is greater than the square of the standardized correlation value of two constructs, discriminate validity is indicated. AVE ranges from 0 to 1, with adequate discriminate validity defined as an AVE greater than 0.50 [60]. The results of composite reliability, AVE, and discriminate validity are reported in Table 1.

## 6. Results

### 6.1. Structural Equation Model

SEM is executed to test the hypothesized structure [61]. Three steps are involved: firstly, the model should achieve satisfactory goodness of fit with the empirical data based on the same set of fit indices applied in assessing the measurement model. Secondly, the direction, significance, and magnitude of the paths corresponding to each hypothesis of the theoretical model are examined. Finally, the squared multiple correlations are examined to determine the proportion of variance, which is explained by the exogenous constructs in the theoretical model.

To validate the model proposed in Figure 1, the SEM technique has been applied for hypothesis testing. The Tucker–Lewis Index, the comparative fit index, and the root mean square error of approximation (RMSEA) are preferred to evaluate the model fitness. The fit indices describe the good fit of the model. The measurement model has confirmed that the items are theoretically close to each other in terms of factor loading and goodness of fit. The model initially tests the with absolute, incremental, and parsimonious measures of fit. Table 2 shows the values of the confirmatory factor analysis of each variable, along with the model of fitness, and Table 3 represents the factor loading results.

### 6.2. Factor Affecting before COVID-19

The proposed model examines with two data sets; participants’ opinions before and during eruption of COVID-19. The consumer responses before COVID-19 revealed that the motivating factors to read food labels have a significant and positive effect on consumer healthy packaged food selection intention. However, the demotivating factors have a significant but negative effect on consumer intention to select healthy packaged food items. Reciprocally, the subjective norms and self-efficacy indicate an insignificant impact on the selection of healthy packaged food. The detailed results are accentuated in Table 4.

### 6.3. Factors Affecting during COVID-19

In comparison to the before COVID-19 opinions, during COVID-19, the subjective norms and self-efficacy have revealed the significant effect of consumer intention to select healthy packaged food. However, the reasons that influence consumer attitudes to avoid food labels also significantly negatively affect their healthy packaged food intentions. The detailed results are indicated by Table 5. Arguably, this attitude has the highest explanatory power, which indicates that during COVID-19 consumers have taken interest in consulting food label information for healthy packaged food selection. It also substantiates the conscious behavior of individuals during COVID-19 towards food selection intention.

## 7. Discussion

The current study contributes to the literature with the specific factors which orientated consumers to select healthy packaged food before and during the eruption of COVID-19. The study has been underpinned by the BRT approach, which is employed by researchers to examine the positive and negative aspects of variables simultaneously. The ultimate results of the study signify that although attitude plays a powerful role in intention, reasons for, and reasons against, it has a noticeable influence on intentions to choose healthy packaged food.

The results of the hypothesized model have identified that all of the hypotheses are significant and supported via empirical evidence. The execution of BRT has demonstrated that consumers’ favorable and unfavorable actions regarding the selection of any object are supported and justified by certain context-specific reasons. Although the current study has not evaluated each reason independently, the overall results demonstrate that BRT contemplates an intention, nevertheless BRT provides context-specific reasons which motivate the individual splendidly towards any object. The perk of BRT is the involvement of context-specific reasons which justify the individual’s behavior towards the acceptance and rejection of any object. Moreover, reasons for attitude and reasons against the attitude is directly affected by intentions. These positive and negative influences demonstrate that there are certain context-specific reasons which facilitate or restrict consumers while taking decisions. During the pandemic, the participants remained in lockdown and were continuously listening to the food recommendations to develop their immune system. Henceforth, the same behavior has been found in their responses in the current study. It is arguable that the reason for attitude has explained the healthy food selection intention with 35%, while the lowest explanatory value was self-efficacy with 2%. Convincingly, it indicates that even during COVID-19, individuals’ internal intentions and motivation to select healthy packaged food has been remained diverted. Reasonably, the factor of this diversion is in accord with the prior studies which illustrate that consumers consume packaged food excessively because of convenience rather than healthiness. In comparison with self-efficacy, the subjective norm has a vigorous effect on the intention to select healthy packaged food during the COVID-19 outbreak. Owing to the intensity of pandemic, the recommendation for the selection of healthy packaged food has also been considered.

It has been observed in previous studies that consumers’ attitudes toward healthy and informed food choices are inconsistent [62]. Similarly, the effect of subjective norms on the intention to use balanced packaged food has not been provided with a comprehensive explanation due to either favorable [63] or unfavorable findings [64]. Previous results regarding self-efficacy’s effect on healthy food selection are also inconsistent. A few researchers have reported that individuals’ internal strength is extremely supportive in taking any decision regarding healthy packaged food, while others have indicated that self-efficacy does not play a role in healthy food choices [65].

The effects of the reasons for and reasons against are perceptible. These reasons indicate that certain context-specific reasons suggest the adoption of FoP label information to enable knowledgeable packaged food selection intention [66,67]. There were nine reasons; health self-consciousness has been signified as the most influential reason. Henceforth, during COVID-19, food processing companies should focus on the health factor of packaged food products while determining food label information. The most decisive context-specific reasons against are the technical information, language difficulty, labels crowded with irrelevant information, the difficulty of interpretation, labels not in the native language, and lack of prior nutritional awareness. The respondents’ feedback on reasons again explains that there are strong reasons that pose a hurdle to consumers successfully interpreting food label information.

People are more conscious about healthy food during COVID-19. Moreover, packaged food is the most preferred food for consumers due to convenience. Henceforth, food processing companies must create awareness among consumers regarding healthy or balanced packaged food consumption.

BRT has assisted the current study in identifying context-specific reasons employing the consumers’ cognitive processes regarding healthy packaged food consumption intentions during COVID-19. Moreover, BRT has also manifested context-specific reasons which influence the intentions of individuals asymmetrically in order to select the healthy packaged foods.

Although this study has unveiled a new dimension for investigating context-specific reasons that play a catalytic role in individuals’ intentions regarding a given subject, there are still certain limitations. For future studies, the researchers should make a list of specific packaged food items while categorizing them to contemplate the usage intensity. In packaged food, there are multiple options and consumers have different behavior towards different packaged food items. Moreover, future studies should contemplate a population having an age range from 18 to 75. The diverse population will provide better results for generalization. Additionally, the proposed model should be examined separately for both genders, demonstrating whether the same reasons for and against can influence both genders.

## 8. Conclusions

This study has employed the BRT technique to investigate the intentions for consuming healthy packaged food. Although BRT originated as an extended form of the theory of planned behavior, it profoundly explains consumers’ intentions regarding any subject. There are certain specific reasons which play a vigorous role in shaping consumers’ specific intentions under the aegis of logical reasoning. Further, these reasons later serve as justifications for decisions. The current study has also revealed certain context-specific reasons that affect consumers’ intentions regarding healthy packaged food selection. Food processing companies employ several label formats to facilitate consumers’ making informed packaged food choices, but these formats are not achieving the desired results. Relevantly, the comprehension of the nutritional information which is printed on food labels varies from consumer to consumer and country to country. Henceforth, the current study has endeavored to identify the specific reasons which provoke consumers to read a food label or restrict them from examining food label information at the point of purchase. Several prior studies have investigated consumers’ intentions regarding food selection but no study has been found which identifies context-specific reasons that influence consumers’ intentions. The extant literature has employed the theory of planned behavior and the theory of reasoned action to investigate intentions, but the BRT technique has emphasized these reasons because human beings need strong justification of their decisions and instinctual behavior. Remarkably, BRT has identified favorable and unfavorable reasons which can assist food processing companies in deciding how to display nutritional information on food labels during COVID-19 while orientating the consumers to select the healthy packaged food.

Perceiving the consumers’ intentions or behavior towards the selection of healthy food is not surprising because several researchers have contributed in various dimensions. Food label formats have remained the main discussion point because there is no formal method for investigating individual intentions about healthy packaged food or restaurants’ food selections. The contribution of the current study is the theory of behavioral reasoning, which facilitates researchers to simultaneously investigate the reasons for and reasons against any object. To the best of our knowledge, no such study has examined consumers’ packaged food intentions with context-specific reasons. This model has provided broader knowledge to food processing companies about consumers in understanding which reasons influence consumers’ intentions in selecting healthy packaged food and which reasons become hindrances in selecting healthy packaged food.

## Figures and Tables

**Table 1:** Composite reliability, average variance extracted and discriminate validity.

Variables	CR	AVE	1	2	3	4	5
Reasonsforattitude	0.765	0.643	0.793				
Reasons against attitude	0.755	0.554	0.184	0.742			
Subjective norm	0.871	0.565	0.263	−0.310	0.801		
Self-efficacy	0.773	0.630	0.172	−0.309	0.309	0.744	
Intention	0.813	0.552	0.296	−0.342	0.407	0.552	0.751

Notes: CR: Composite Reliability, AVE: Average Variance Extraction.

**Table 2:** Confirmatory factor analysis and model fitness.

Code	Items	Chi-S	CMIN	CFI	GFI	AGFI	NFI	RMSEA	P-V
Reason for attitude	9	7.522	1.761	0.997	0.997	0.980	0.990	0.038	0.172
Reason against attitude	9	8.404	1.681	0.996	0.994	0.981	0.990	0.038	0.135
Intention	7	2.216	1.108	0.998	0.999	0.990	0.995	0.014	0.330
Subjective norm	7	4.019	2.010	0.998	0.996	0.981	0.996	0.043	0.134
Self-efficacy	9	8.478	1.696	0.994	0.990	0.982	0.976	0.036	0.132
Indicators	Hypothesized Model			Threshold Values (Hair et al., 2010)					
Absolute									
Chi-Square	156.676			Less than 2					
DF	124								
Ratio/CMIN	1.276								
Incremental									
CFI	0.992			Greater than 0.90					
GFI	0.969			Greater than 0.90					
AGFI	0.956			Greater than 0.90					
NFI	0.952			Greater than 0.90					
Parsimonious									
RMSEA	0.019			Less than 0.080 (lesser is better)					
P-value	0.059			Greater than 0.05 (bigger is better)					

**Table 3:** Factors loading.

Constructs	Item	Loading
Subjective Norms	SN1	0.647
	SN2	0.832
	SN3	0.728
	SN4	0.733
Self-Efficacy	SE5	0.741
	SE2	0.828
	SE3	0.715
	SE6	0.768
Reasons for Attitude	RFA3	0.810
	RFA5	0.794
	RFA6	0.778
	RFE7	0.674
Reasons Against Attitude	RAA4	0.605
	RAA5	0.818
	RAA7	0.847
	RAA9	0.792
Healthy Packaged food Intention	PI3	0.850
	PI5	0.773
	PI6	0.804

**Table 4:** Standardized results before COVID-19.

Endo		Exog	Estimate	S.E.	C.R.	P	Status
Intention	<---	Reason for Attitude	0.156	0.036	1.935	0.005	Significant
Intention	<---	Reason Against Attitude	−0.056	0.043	2.423	0.002	Significant
Intention	<---	Self-Efficacy	0.047	0.201	−0.146	0.070	Insignificant
Intention	<---	Subjective norm	0.210	0.234	0.727	0.080	Insignificant

**Table 5:** Standardized results during COVID-19.

Endo		Exog	Estimate	S.E.	C.R.	P	Status
Intention	<---	Reason for Attitude	0.356	0.088	2.145	0.036	Significant
Intention	<---	Reason Against Attitude	−0.256	0.070	2.316	0.026	Significant
Intention	<---	Self-Efficacy	0.027	0.106	−0.253	0.030	Significant
Intention	<---	Subjective norm	0.121	0.197	0.613	0.040	Significant

## Data Availability

The data that support the findings of this study are available from the corresponding authors upon reasonable request.

## Appendix A

**Table A1:** Measurements.

**Reasons for consulting Food label**	**1**	**2**	**3**	**4**	**5**
Usefulness					
Ease of interpretation					
Visual salience					
Familiarity with symbols					
Parental awareness					
Health self-consciousness					
Relevant information					
Acquisition of nutritional knowledge					
Social responsibility					
**Reasons against consulting Food label**	**1**	**2**	**3**	**4**	**5**
Technical information					
Language difficulty					
Description of nutritional benefits with unclear symbols					
Label crowded with irrelevant information					
Label information is not trustworthy					
Food labels are only advertising strategies					
Difficulty of interpretation					
Not in native language					
Lack of prior nutritional awareness					
**Subjective Norms**					
People important to me think I should eat healthy package food	**1**	**2**	**3**	**4**	**5**
People important to me approve to eat healthy package food	**1**	**2**	**3**	**4**	**5**
People important to me want me to eat healthy package food	**1**	**2**	**3**	**4**	**5**
Many people who are important to me eat healthy package food	**1**	**2**	**3**	**4**	**5**
The mass media suggest that I should use healthy package food products	**1**	**2**	**3**	**4**	**5**
The mass media urge me to use healthy package food products	**1**	**2**	**3**	**4**	**5**
The mass media and advertising consistently recommended that I should use healthy package food products	**1**	**2**	**3**	**4**	**5**
**Self-Efficacy**					
For me it is difficult to select healthy package food due to small font size at a food label.	**1**	**2**	**3**	**4**	**5**
For me it is difficult to select healthy package food due to lack of knowledge about nutrients.	**1**	**2**	**3**	**4**	**5**
My nature to eat quickly hinders me to select healthy package food.	**1**	**2**	**3**	**4**	**5**
It is entirely up to me to select healthy package food	**1**	**2**	**3**	**4**	**5**
Shopping foods with others (e.g., friends) make difficult for me to select healthy package food	**1**	**2**	**3**	**4**	**5**
For me it is difficult to select healthy package food because nutritional information is placed at the back of the pack food label	**1**	**2**	**3**	**4**	**5**
It is easy to select healthy package food if I can understand the nutrients on the label (e.g., Calorie, fat, etc.).	**1**	**2**	**3**	**4**	**5**
It is easy to select healthy package food if I can understand the nutrient content per serving size on the label (e.g., Calorie 400 kcal, fat 10 g, etc.)	**1**	**2**	**3**	**4**	**5**
It is easy to select healthy package food if I can understand the percentage daily values of nutrients on the label	**1**	**2**	**3**	**4**	**5**
**Healthy packaged food Intention**	**1**	**2**	**3**	**4**	**5**
I give importance to nutrients in the purchasing of packaged food items	**1**	**2**	**3**	**4**	**5**
I mostly prefer to eat healthy package food	**1**	**2**	**3**	**4**	**5**
I frequently purchase healthy package food	**1**	**2**	**3**	**4**	**5**
I am willing to pay extra for healthy package food	**1**	**2**	**3**	**4**	**5**
I intend to take healthy package food	**1**	**2**	**3**	**4**	**5**
I plan to take healthy package food	**1**	**2**	**3**	**4**	**5**
I want to take healthy package food	**1**	**2**	**3**	**4**	**5**
